# Fusing Data Mining, Machine Learning and Traditional Statistics to Detect Biomarkers Associated with Depression

**DOI:** 10.1371/journal.pone.0148195

**Published:** 2016-02-05

**Authors:** Joanna F. Dipnall, Julie A. Pasco, Michael Berk, Lana J. Williams, Seetal Dodd, Felice N. Jacka, Denny Meyer

**Affiliations:** 1 IMPACT Strategic Research Centre, School of Medicine, Deakin University, Geelong, VIC, Australia; 2 Department of Statistics, Data Science and Epidemiology, Swinburne University of Technology, Melbourne, VIC, Australia; 3 Department of Medicine, The University of Melbourne, St Albans, VIC, Australia; 4 Department of Epidemiology and Preventive Medicine, Monash University, Melbourne, VIC, Australia; 5 University Hospital Geelong, Barwon Health, Geelong, VIC, Australia; 6 Department of Psychiatry, The University of Melbourne, Parkville, VIC, Australia; 7 Florey Institute of Neuroscience and Mental Health, Parkville, VIC, Australia; 8 Orygen, the National Centre of Excellence in Youth Mental Health, Parkville, VIC, Australia; 9 Centre for Adolescent Health, Murdoch Children’s Research Institute, Melbourne, Australia; 10 Black Dog Institute, Sydney, Australia; Qom University, ISLAMIC REPUBLIC OF IRAN

## Abstract

**Background:**

Atheoretical large-scale data mining techniques using machine learning algorithms have promise in the analysis of large epidemiological datasets. This study illustrates the use of a hybrid methodology for variable selection that took account of missing data and complex survey design to identify key biomarkers associated with depression from a large epidemiological study.

**Methods:**

The study used a three-step methodology amalgamating multiple imputation, a machine learning boosted regression algorithm and logistic regression, to identify key biomarkers associated with depression in the National Health and Nutrition Examination Study (2009–2010). Depression was measured using the Patient Health Questionnaire-9 and 67 biomarkers were analysed. Covariates in this study included gender, age, race, smoking, food security, Poverty Income Ratio, Body Mass Index, physical activity, alcohol use, medical conditions and medications. The final imputed weighted multiple logistic regression model included possible confounders and moderators.

**Results:**

After the creation of 20 imputation data sets from multiple chained regression sequences, machine learning boosted regression initially identified 21 biomarkers associated with depression. Using traditional logistic regression methods, including controlling for possible confounders and moderators, a final set of three biomarkers were selected. The final three biomarkers from the novel hybrid variable selection methodology were red cell distribution width (OR 1.15; 95% CI 1.01, 1.30), serum glucose (OR 1.01; 95% CI 1.00, 1.01) and total bilirubin (OR 0.12; 95% CI 0.05, 0.28). Significant interactions were found between total bilirubin with Mexican American/Hispanic group (p = 0.016), and current smokers (p<0.001).

**Conclusion:**

The systematic use of a hybrid methodology for variable selection, fusing data mining techniques using a machine learning algorithm with traditional statistical modelling, accounted for missing data and complex survey sampling methodology and was demonstrated to be a useful tool for detecting three biomarkers associated with depression for future hypothesis generation: red cell distribution width, serum glucose and total bilirubin.

## Background

Over the last two decades there has been a steady rise in the use of data mining techniques across a number of disciplines. Data mining incorporates a path to knowledge discovery and is a meaningful process for discovering patterns in data by exploring and modelling large quantities of data [1, 2]. The distinction between statistics and data mining has been attributed to the nature of the analysis; statistics deals with primary analysis, whereas data mining deals with secondary analysis [3] that learns from data [4]. Data mining incorporates machine-learning algorithms to learn, extract and identify useful information and subsequent knowledge from large databases [2].

For a number of years, data mining techniques incorporating machine learning algorithms have been used for ‘big data’ analytics in both marketing and finance (i.e. cost saving, sales opportunity) [5]. Supervised machine learning techniques have been successfully used in these industries for feature or variable reduction to produce highly predictive models [6]. However, it has only been over the last 10 years that data mining techniques have been used in medical research, primarily in neuroscience and biomedicine [7, 8]. More recently, psychiatry has begun to utilize the benefits of these techniques to gain further insight into the genetic makeup of mental illness [9]. However, the implementation of data mining techniques in large epidemiological studies has not been fully explored.

Epidemiological observational studies are based on a particular study population, often followed over a period of time, and usually involving no intervention other than the administration of questionnaires and the carrying out of medical and laboratory or biomarker examinations. These studies have been used to quantify prevalence and risk factors for diseases within the population [10]. Sample sizes for these types of studies often comprise of some thousands of individuals. Incorporating a methodology involving data mining techniques using a machine learning algorithm with traditional statistics for variable selection in these studies would augment the effective knowledge discovery processes of data mining and rigors of machine learning algorithms with the well-established metrics of traditional statistics. A hybrid methodology as such could take account of common analytical issues associated with these types of studies, such as large numbers of variables, a complex survey design and missing data. For these reasons, the aim of this study was to develop a systematic and sound hybrid methodology involving these elements to perform variable selection that would be appropriate for use in large epidemiological studies.

To test the proposed hybrid methodology, data from a large cross section population based U.S. epidemiology study was utilised to identify key biomarkers for depression. Depression is a serious medical illness, with the World Health Organization estimating that 350 million people worldwide are affected by depression, with depressive disorders ranking second in terms of global disability burden and depression expected to be the number one health concern in both developed and developing nations by 2020 [11–13].

Biomarkers are used in medicine as indicators of risk, diagnosis or trait, disease state or acuity, stage of illness, treatment response and prognosis [14]. Identifying diagnostic biomarkers of depression may help in its detection and furthermore, circumvent the onset. Despite intensive approaches into the investigation of biomarkers in psychiatry, limitations of sensitivity and specificity of single biomarkers have made it impractical to assess an individual’s clinical situation and make determinations regarding diagnosis and prognosis on the basis of biomarkers. Thus, establishing key informative biomarkers would be of benefit to psychiatry [15]. The nature of this type of data is such that missing data often needs to be accommodated using multiple imputation and the complex survey sampling methodology taken into account. To date, conventional variable selection methods do not deal with both these issues associated with this type of data.

The proposed novel hybrid methodology involved a three-step approach, combining data mining techniques using the machine learning algorithm of boosted regression and bagging, with traditional statistical techniques. To take into account missing data and complex survey sampling methodologies, this new three-step approach to variable selection was developed. This methodology was effectively used to identify key biomarkers for depression in a large cross-sectional population-based U.S. epidemiological study.

## Methods

### Study design and participants

Data from the National Health and Nutrition Examination Survey (NHANES) (2009–2010) [16] were utilised. Relevant NHANES data files were downloaded from the website and integrated using the Data Integration Protocol In Ten-Steps (DIPIT) [17]. NHANES is a cross-sectional, population-based study of approximately 10,000 non-institutionalised U.S. civilians aged 18 to 80 years, conducted in two-year blocks. A four-stage sampling methodology was applied: counties; segments within counties; households within segments; and finally, individuals within households. Data were collected from 15 different locations across 50 states of America and the District of Columbia. In addition, oversampling of subgroups of the population of particular public health interest was performed to increase the reliability and precision of population estimates [16]. Finally, subsamples for the mental health and laboratory components of the survey were chosen, at random, with a sampling frame especially designed to reduce respondent fatigue and help scheduling in the biomarker collection [16, 18].

Over 250 biomarkers were available for 5,546 participants. Duplicate biomarkers (n = 75), biomarkers with a high missing count predominantly due to the sampling protocol (n = 94) and low incidence (n = 26) were excluded from the analysis. The final set consisted of 67 biomarkers for inclusion. Of the 5,546 participants, 5.6% were excluded from the analysis due to having six or more missing data values across the 67 biomarkers, and a further six outlier cases were removed to enable the multiple imputation to converge. The final sample size included in this research study was 5,227.

NHANES received approval from the National Center for Health Statistics (NCHS) research ethics review board and informed consent was obtained for all participants. Use of data from the NHANES 2009–2010 is approved by the National Center for Health Statistics (NCHS) Research Ethics Review Board (ERB) Approval for NHANES 2009–2010 (Continuation of Protocol #2005–06).

### Study Measurements

The Patient Health Questonnaire-9 (PHQ-9) [19] was used to assess depressive symptoms (depression). The PHQ-9 is a well-validated, self-report tool for detecting and monitoring depression, with good concordance with a clinical diagnosis of major depressive disorder (MDD) [20]. Items assess the presence of nine Diagnostic and Statistical Manual of Mental Disorder Fourth Edition (DSM-IV) depression symptoms over the past two weeks, and are scored on a four-point scale indicating the degree of severity from 0 (not at all) to 3 (nearly every day). Items were then summed to form a total severity score ranging from 0 to 27 where those with a total score of 10 or more were considered depressed (i.e. moderately to severely depressed) [21].

Health report status was measured as an ordinal five-point rating scale (excellent, very good, good, fair, poor) and incorporated as predictor in the multiple imputation models as an indicator of general health.

Blood and urine samples were collected in the Mobile Examination Center (MEC) and shipped weekly for laboratory analyses. Specific laboratory techniques for each test are available from the NHANES Laboratory Procedures Manual [22].

Demographic variables were self-reported and included: age, gender, and race (collapsed into four groups: Mexican American and other Hispanic, Non-Hispanic White, Non-Hispanic Black, and other).

Current and past smoking status was determined from self-reported questions from the smoking cigarette use component from NHANES. Smoking status was categorized into those who were never, former or current smokers. Physical activity was grouped into active (low to high activity) and not active from the physical activity component from NHANES. Alcohol regularity per month was calculated from the self-report question regarding how often alcohol was drunk over the past 12 months, calculated from the weekly, monthly and yearly figures.

Food insecurity, a measure relating to the limited or uncertain access to food due to inadequate financial resources, was determined from the 10-item Food Security Survey Module (FSSM) [23] and categorized into two groups: full food security (no affirmative response in any of the 10 items); and food insecurity (at least one affirmative responses).

Body measurements were collected in the MEC by trained health technicians. Weight and height were measured and body mass index (BMI) calculated as weight/height^2^ (kg/m^2^). BMI was categorised as underweight (BMI < 18.5 kg/m^2^), normal (BMI between 18.5 to under 25 kg/m^2^), overweight (between 25 to under 30 kg/m^2^) and obese (BMI ≥30kg/m^2^) [24].

Participants were identified as having Type 2 diabetes if they (i) reported that either a doctor or health professional had told them they had diabetes or sugar diabetes (other than during pregnancy); (ii) reported the use of hyperglycaemic agents, with the medication bottle seen by the interviewer [25]; and/or (iii) diabetes based on their fasting blood glucose >126 mg/dL (7.0 mmol/L) [26–29] and glycated haemoglobin level of >6.5% [30–32].

Participants were identified as having cardiovascular disease using the self-report questions regarding past history of heart conditions (i.e. congestive heart failure, coronary heart disease, angina/angina pectoris, heart attack, stroke) which have been shown to have high concordance with laboratory measures for cardiovascular disease [33, 34].

Participants were also identified as having the inflammatory conditions of arthritis and cancer or malignancy using the self-report questions.

Participants were asked to bring currently used medications to the assessment. The Lexicon Plus^®^ proprietary comprehensive database of Cerner Multum, Inc. was used to assist with data collection, data editing and release of the NHANES medication data. All prescription and some non-prescription drug products available in the U.S. were coded using the Lexicon Plus medication database. For the current analyses, Lexicon Plus medication categories with higher than 2% incidence were included: anti-infective, cardiovascular agents, central nervous system agents, coagulation modifiers, gastrointestinal agents, hormones/hormone modifiers, metabolic agents, nutritional products, psychotherapeutic agents, respiratory agents, and topical agents.

## Methodology

**Fig 1:**
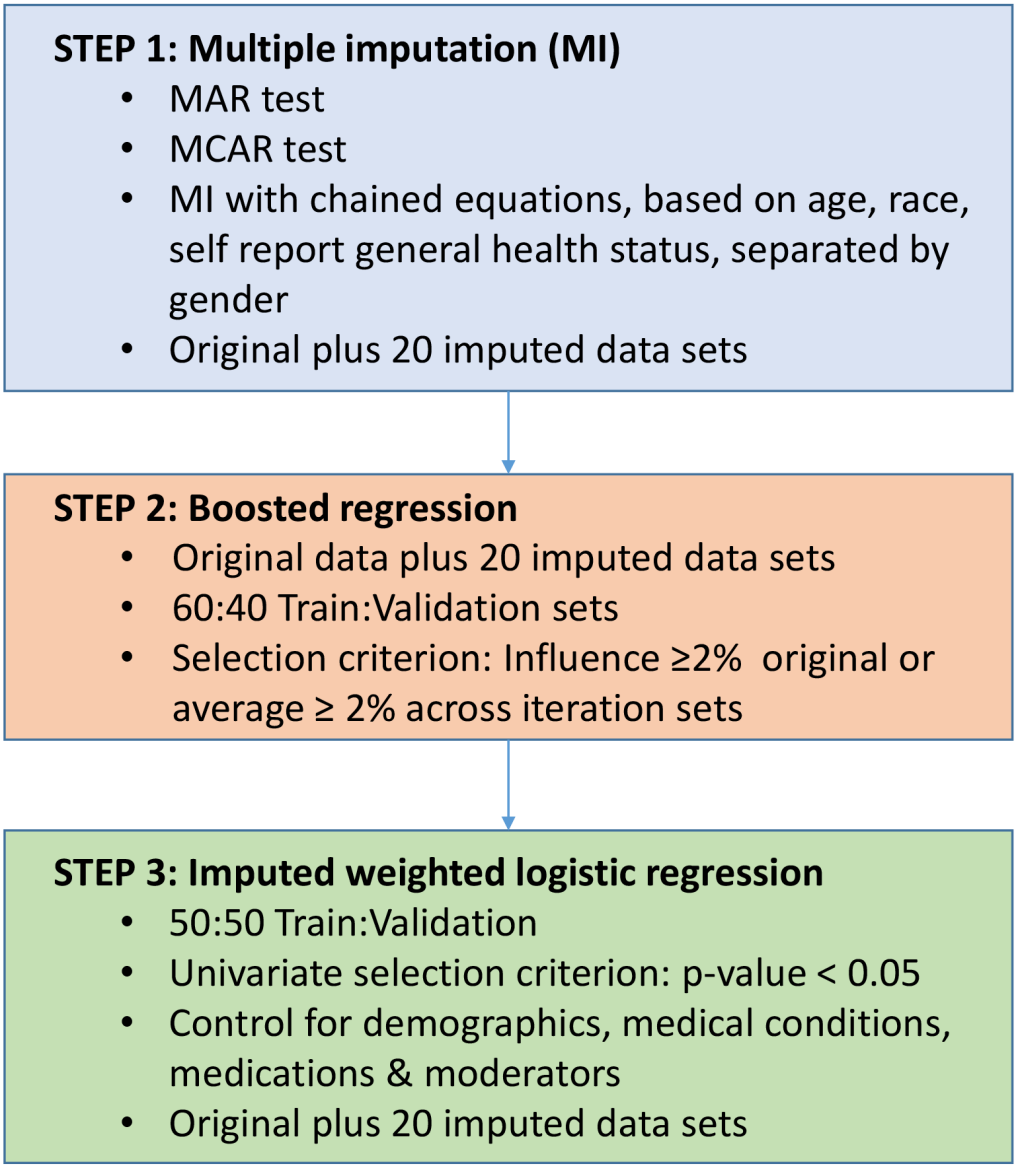
Hybrid Methodology Steps.

The proposed hybrid methodology was applied to this study to select key biomarkers associated with depression from the NHANES data set (Fig 1).

### STEP 1: Multiple Imputation (MI) for Missing Data

In many ‘big data’ situations, missing data are not an issue due to the large volume of observations and variables or features available. In contrast, missing data in studies with small sample sizes can influence the results greatly [35, 36]. There have been a number of missing data procedures suggested in the literature over the last decades: listwise deletion; pairwise deletion; mean substitution; regression imputation; Maximum Likelihood (ML) estimation [37, 38]. However, most of these methods can only be used when there is no pattern for the missing data. The choice of method used for dealing with missing data is often less important when the proportion of missing data is less than 5% [39]. However, it is not unusual for the proportion of missing data in large epidemiological studies to exceed this percentage, thereby potentially reducing statistical power, producing biased parameters and increasing the risk of a Type I error [35]. All these issues could be detrimental to traditional statistical multivariate regression models.

Multiple imputation is a useful flexible strategy for addressing the missing value problem. Multiple imputation is considered when the missingness is not totally random, depending on observed or unobserved values. However, this method is applicable even when the pattern of missing data is not random. Multiple imputation using chained sequences of equations is a flexible imputation method with the ability to handle different variable types, as an imputation model is generated for each variable with missing data [40]. This method is also referred to as fully conditional specification [41] and sequential regression multivariate imputation [42]. A selected imputation model generates ‘mi’ complete imputed sets of data. Analysis is then performed and results are pooled. The number of ‘mi’ sets depends on the amount of missing data, nature of the data and analysis model used. More than 50 imputed sets may be required to obtain stable results [43], but between 5 to 20 has been considered appropriate to reduce the imputation sampling error when fractions of missing data are low [44].

Prior to imputing the missing data, missing values are often tested for Missing At Random (MAR) or Missing Completely At Random (MCAR) [38, 45]. MAR occurs when the probability of being missing is dependent on the variables measured in the study, but independent of those not measured in the study. To test MAR, logistic regression is often performed with a missing data indicator created for each potential predictor (i.e. 1 for missing, 0 for non-missing). No significant relationship between the missingness indicators and the outcome of interest suggests MAR. MCAR occurs when the probability of being missing is independent of all the variables in the study (those measured and not measured) The assumption that the data are missing completely at random (MCAR) can be assessed using Little's MCAR chi-squared test, which can be applied to a large set of variables [46, 47], including all the predictors simultaneously. A significant result rejects the null hypothesis that the data is MCAR. Rejection of MAR or MCAR confirms the need for multiple imputation for the missing values in the data.

As the highest missing data percentage for this study data set was above 5% and the assumptions of MAR and MCAR were rejected, multiple imputation was required. The multiple imputation framework of inference was developed by Rubin [38] and implemented in Stata. Multiple imputation was performed, with chained sequences of equations [40, 48] using all biomarkers, age, race, and a measure of self-report health status, but run separately for males and females due to possible differences in biomarker importance between the sexes [49]. A mix of binary logistic and linear regression, using chained equations, was used, contingent on the nature of the biomarker imputed. This study involved 20 chained sequences with the primary data set of 5,227 observations. Thus, the combined original and imputed data sets contained 109,767 observations. Convergence for each of the chained equations was achieved within an acceptable 20 imputed data sets [44].

### STEP 2: Technique for Initial Selection of Predictors (Boosted Regression)

There are many potential statistical and machine learning algorithms available for variable selection [2], but boosted regression is recognised as being particularly effective [50]. This technique has often been thought of as a ‘bucket’ or ensemble method (a method that averages over multiple classifiers), and has gained popularity amongst mainstream statisticians due to its predictive accuracy and ease of interpretation [51, 52]. Boosted regression has been used in studies involving animal ecology [53], for example to build predictive models for fish and coral metrics [54], and to identify factors predicting the reproductive success of dominant pairs of clown anemonefish [55]. It has also been used in the development of the Chicago Adolescent Depression Risk Assessment (CADRA) index from an array of baseline social and cognitive vulnerability and mood risk factors [56].

Boosting was invented by Freund and Schapire for regression trees [57] and translated to the logistic regression model by Hastie, Tibshirani, and Friedman [54]. One of the advantages with boosted regression is that the algorithm can accommodate any type of variable (continuous, categorical, censored), any type of likelihood loss function (Gaussian, Binomial, Poisson, and robust), and can deal with highly correlated predictors [58]. In addition it can automatically accommodate non-linearity and interaction effects between predictor variables. The boosting regression algorithm fits a sequence of simple trees based on a set of data splitting rules to provide a more accurate estimate of the outcome with over-fitting avoided using a validation data set and shrinkage. Each tree consists of a series of yes/no questions which is applied to each observation in the training data. Predictions are obtained using the average of the outcomes for the observations found in each terminal node. Residuals are computed as the difference between the true and outcome values. Each successive tree is built for the prediction of the residuals of the preceding tree. Essentially the technique successively gives larger weights to observations that are repeatedly misclassified.

The final classifier consists of a weighted average of previous classifiers, with lower weights (shrinkage) for later trees in order to avoid over-fitting. Shrinkage is accomplished by adding a parameter λ to the last regression tree of residuals, where λ = 1 corresponds to no shrinkage. Typically λ is 0.1 or smaller, with λ = 0.01 or λ = 0.001 common. Smaller shrinkage values require larger number of iterations which can be computationally demanding.

The steps in the boosted regression technique incorporating the shrinkage parameter:

- An initial guess for the predicted outcomes is created. Compute the residuals based on the current model.
- For each regression tree:
  - Fit a regression tree to the residuals
  - Compute the predicted outcome of the residuals for each terminal node
  - Use the regression tree to predict the new set of residuals
  - Update the boosting regression model to reflect the current regression tree, applying the shrinkage parameter to the last regression tree of residuals
  - This model is applied to the validation data and if there is an improvement in predictive accuracy over the results for the previous iteration the next iteration commences. If not, then training stops and no more iterations are executed and no more residual trees are developed.

In order to improve the robustness of the final model without introducing bias ‘bagging’ was used [59]. Bagging is sometimes referred to as bootstrap aggregating and was originally developed by Breiman as a method for creating multiple versions of a predictor to form an aggregated predictor [60]. Combining this method with boosting, bagging randomly selects a proportion of the residuals at each iteration to build a new tree. While not all observations are used in each iteration, all observations are eventually used across all iterations. Thus, the tree analysis is run on multiple similar datasets, and the aggregate results are used to construct the final model and relevant statistics. Friedman recommends bagging with 50% of the database [59].

The final boosted regression tree model tends to be more robust than a single regression tree model, enabling complex functions and interactions to be modelled without assumptions [53]. Since no probability values (p-value) are produced from the boosted regression algorithm, the relative importance of variables has been used to pick likely predictors [59]. Each variable is assigned a relative importance percentage or contribution, where the sum of the standardized values of all variable importances add to 100%. The importance is calculated from the number of times a variable is selected for splitting, weighted by the squared improvement to the model fit achieved from each such split, averaged over all trees. Higher values of importance indicate stronger contributions to outcomes values. With a binary outcome measure the importance represents the percentage of the log likelihood explained by each variable. For the proposed methodology, the importance percentage for each predictor will be of particular interest for the variable selection process.

The analysis approach for selecting the preliminary subset of predictors for the proposed methodology is one of inclusion rather than exclusion. A potential predictor was selected based on the relative importance percentage in the original data set and the average importance percentage statistics across the 20 imputed data sets. The cut-off relative importance percentage is therefore relatively inclusive, ensuring that a reasonable percentage of the total log likelihood is retained.

For the NHANES study, depression was a binary indicator. The boosted regression method used the Multiple Additive Regression Trees (MART) boosting algorithm [54, 59] implemented by Schonlau [61]. This method was performed on the original data set of 5,227 observations with missing data, then for each of the 20 imputed data sets. For each run, validation was performed by randomly splitting each data set into 60% training and 40% validation ensuring that additional boosting iterations were employed only when improved prediction accuracy was achieved with the validation data set. After some initial testing, the final shrinkage parameter used was 0.001 with the recommended 50% of the residuals used to fit each individual tree (50% bagging) [59]. The maximum number of boosting interactions (i.e. number of terminal nodes plus 1) allowed was 6, being marginally higher than the default (i.e. 5) and within the recommended range by Hastie, Tibshirani and Friedman [54]. Finally, a random-number seed was used to generate the same sequence of random numbers for bagging, ensuring that the results could be reproduced. Biomarkers with the greatest relative importance for the prediction of depression were identified.

To ensure the inclusion criterion was implemented and a reasonable proportion of the total relative importance was retained, cut-off percentages of 4%, 3% and 2% were tested. It was decided to use 2% to ensure more than 50% of the total relative importance was included in this stage of variable selection. Thus, a biomarker was included in the initial biomarker selection if its relative importance percentage in the original data set was higher than 2%, or its average importance percentage statistics across the 20 imputed data sets yielded average of at least 2%.

### STEP 3: Traditional statistical regression

At the heart of data mining is the concept of data splitting to address overfitting the data from the machine learning techniques used. Data are randomly split into two groups: a training set and a validation set. Thus, the imputed data set was split into training and validation data sets. For the NHANES study, the imputed data set was split into approximately 50% training and 50% validation data for the traditional statistical analysis. Each set maintained the balance in regard to depression levels and imputation sets.

The standard approach of univariate regressions for each of the potential predictors selected from step two were then performed, with the variable of interest as the outcome variable. Predictors with the strongest relationship with the outcome for both these data sets were chosen based on traditional statistical 95% confidence (i.e. p<0.05). As required for the NHANES data, the study’s complex survey sampling methodology was taken into account using univariate weighted logistic regressions. These regressions, were conducted for each selected biomarker from the machine learning boosted regression algorithm with depression as the outcome. Biomarkers having a significant relationship with depression were chosen (i.e. p<0.05) at the final variable selection stage.

Using the selected biomarkers from the univariate analysis, a traditional statistical multivariate regression model was then estimated for both the training and validation data sets. Multicollinearity and mediation relationships were tested and predictors removed accordingly. For the NHANES data, it was important that this step took account of the complex four-staged survey design.

Finally, demographic and medical covariates and significant interactions were included in this multivariate model to control for any confounders and account for any moderation effect with models fitted using both the training and validation imputed data sets. The final model was also fitted using the original set of observations (with missing values) to obtain an indicative measure for goodness of fit and to ensure consistent direction and significance for the important biomarkers identified using the primary data set and the combined data set with imputation for missing values.

All statistical procedures were performed using Stata V14 software (StataCorp., 2014). A Stata plugin was used for the boosted regression component of the analysis [61].

## Results

Estimated statistics for each of the covariates and the significance of the relationship with depression for each covariate is presented in Table 1. The complex survey design of NHANES is taken into account in these estimates.

**Table 1:** Estimated covariate statistics.

**Covariate**	**Proportion**	**Std Error**	**95% CI Low**	**95% CI High**	**p-value**
**Depressed**					
Not Depressed	0.923	0.005	0.913	0.934	
Depressed	0.077	0.005	0.066	0.087	
**Gender**					
Male	0.496	0.006	0.483	0.509	Reference
Female	0.504	0.006	0.491	0.517	<0.001
**Age Group**					
18–34 years	0.329	0.009	0.309	0.348	Reference
35–44 years	0.210	0.008	0.193	0.226	0.457
45–54 years	0.223	0.007	0.207	0.238	0.117
55–64 years	0.176	0.006	0.162	0.189	0.272
65+ years	0.063	0.004	0.056	0.071	0.006
**Race**					
Mexican American/Hispanic	0.147	0.031	0.082	0.213	0.010
Non-Hispanic White	0.678	0.035	0.602	0.753	Reference
Non-Hispanic Black	0.110	0.009	0.089	0.130	<0.001
Other	0.065	0.009	0.046	0.084	0.542
**Smoking**					
Current Smoker	0.219	0.008	0.201	0.237	<0.001
Former Smoker	0.226	0.014	0.195	0.257	0.800
Never Smoked	0.555	0.019	0.515	0.595	Reference
**Food Security**					
Full food security	0.782	0.013	0.755	0.809	<0.001
Food insecurity	0.218	0.013	0.191	0.245	Reference
**BMI Category**					
Underweight	0.018	0.003	0.012	0.024	0.396
Normal	0.295	0.014	0.266	0.324	Reference
Overweight	0.331	0.011	0.307	0.355	0.358
Obese	0.356	0.011	0.333	0.379	0.035
**Physical Activity**					
Low to high activity	0.451	0.018	0.411	0.490	Reference
No low to high activity	0.549	0.018	0.510	0.589	0.882
**Diabetes Status**					
No Diabetes	0.910	0.005	0.900	0.920	Reference
Diabetes	0.090	0.005	0.080	0.100	0.001
**Cardiovascular Disease Status**					
No Cardiovascular Disease	0.924	0.007	0.910	0.938	Reference
Cardiovascular Disease	0.076	0.007	0.062	0.090	0.009
**Arthritis Status**					
No Arthritis	0.928	0.005	0.918	0.938	Reference
Arthritis	0.072	0.005	0.062	0.082	<0.001
**Cancer or malignancy**					
No Cancer or malignancy	0.901	0.008	0.884	0.919	Reference
Cancer or malignancy	0.099	0.008	0.081	0.116	0.925
**Use Central Nervous System**					
No	0.854	0.008	0.836	0.871	Reference
**Medication(s)**					
Yes	0.146	0.008	0.129	0.164	<0.001
**Use Psychotherapeutic Agents**					
No	0.915	0.006	0.902	0.928	Reference
Yes	0.085	0.006	0.072	0.098	<0.001
	**Mean**	**Std Error**	**95% CI Low**	**95% CI High**	**p-value**
**Family Poverty Income Ratio (PIR)**	3.013	0.044	2.918	3.108	<0.001
**Number of times drink alcohol pm**	6.235	0.290	5.615	6.856	0.005

Note: Multiple-imputation, survey estimation. Based on 20 imputations, primary N = 5,227. P-value indicates the significance of biomarker with depression.

### STEP 1: Multiple Imputation results

The impact of missing data across the 67 laboratory data was quantified [17]. The results from the imputed weighted logistic regression with the missing data indicator for each biomarker as the outcome and depression as the predictor are reported in the last column in Table 1. Significant results for several biomarkers indicated that missing biomarker data affected the depression status (i.e. p<0.05), confirming that the data was not MAR. Little's test also provided evidence against the assumption that all laboratory data were MCAR (p<0.001). The missing data were therefore imputed using multiple imputation.

### STEP 2: Machine Learning boosted regression results

Multiple imputation produced 20 separate data sets with distinct imputed missing values for the boosted regression step. The relative importance statistics are reported in Table 2 for the original data set and across the 20 imputed data sets. Based on the selection criterion explained above, 21 biomarkers were selected for the next stage of the analysis. The selected biomarkers consistently explained more that 50% of the total relative importance for both the original data set (53.85%) and for the mean values computed across the 20 imputed data sets (53.33%).

**Table 2:** Boosted regression statistics.

Biomarker	Original data set		Imputation	sets 1 to 20	
		Mean	Std Dev	Min	Max
T.gondii antibodies (IU/ml)	0.220	0.562	0.444	0.326	2.388
Blood lead (ug/dL)	1.482	1.658	0.066	1.537	1.753
Mercury, total (ug/L)	1.958	1.847	0.110	1.628	2.049
Mercury, inorganic (ug/L)	0.290	1.668	0.116	1.358	1.788
White blood cell count (1000 cells/uL)	1.243	1.126	0.065	1.000	1.277
Lymphocyte percent (%)	1.331	0.978	0.078	0.780	1.176
Monocyte percent (%)	1.996	1.595	0.172	1.371	1.904
Segmented neutrophils percent (%)	1.240	1.004	0.082	0.856	1.121
Eosinophils percent (%)	1.770	0.971	0.126	0.819	1.406
Basophils percent (%)	0.565	0.585	0.051	0.503	0.690
Lymphocyte number (1000 cells/uL)	0.754	0.912	0.171	0.691	1.559
Monocyte number (1000 cells/uL)	0.835	0.492	0.043	0.392	0.552
Segmented neutrophils num (1000 cell/uL)	1.138	1.103	0.075	0.950	1.282
Eosinophils number (1000 cells/uL)	0.229	0.129	0.026	0.052	0.157
Basophils number (1000 cells/uL)	0.069	0.084	0.012	0.056	0.100
Red blood cell count (million cells/uL)	0.829	1.083	0.148	0.898	1.595
**Hemoglobin (g/dL)**	1.717	**2.942**	0.152	2.667	3.210
**Hematocrit (%)**	0.847	**2.798**	0.148	2.533	3.103
Mean cell volume (fL)	0.494	0.943	0.082	0.853	1.154
Mean cell hemoglobin (pg)	0.848	1.307	0.045	1.251	1.432
**Mean Cell hemoglobin concentration (MCHC) (g/dL)**	**5.139**	**3.380**	0.105	3.195	3.610
**Red cell distribution width (%)**	**3.423**	1.918	0.105	1.685	2.058
**Platelet count (1000 cells/uL)**	**2.395**	**2.632**	0.112	2.443	2.842
Mean platelet volume (fL)	1.257	1.348	0.095	1.214	1.519
**Blood cadmium (nmol/L)**	**5.306**	**4.159**	0.136	3.908	4.447
Glycohemoglobin (%)	1.526	1.830	0.107	1.604	1.986
**C-reactive protein(mg/dL)**	1.877	**2.264**	0.107	2.024	2.405
Direct HDL-Cholesterol (mg/dL)	1.097	1.149	0.104	0.946	1.328
RBC folate (ng/mL)	0.536	0.779	0.097	0.588	1.021
Serum folate (ng/mL)	1.955	1.840	0.163	1.486	2.087
**Cotinine (ng/mL)**	**2.011**	1.982	0.277	1.542	2.739
**Urinary Total NNAL (ng/mL)**	**3.226**	**5.579**	0.496	4.544	6.459
Albumin (g/dL)	0.914	0.543	0.050	0.459	0.662
Alanine aminotransferase ALT (U/L)	0.843	0.836	0.033	0.791	0.889
Aspartate aminotransferase AST (U/L)	0.727	0.577	0.053	0.462	0.694
**Alkaline phosphotase (U/L)**	**2.992**	1.966	0.086	1.825	2.184
Blood urea nitrogen (mg/dL)	0.840	0.875	0.088	0.718	1.061
Total calcium (mg/dL)	0.671	0.610	0.053	0.528	0.753
Cholesterol (mg/dL)	0.373	0.793	0.086	0.680	1.076
**Bicarbonate (mmol/L)**	**3.769**	1.840	0.089	1.661	2.035
**Creatinine (mg/dL)**	**3.286**	**3.041**	0.189	2.491	3.370
Gamma glutamyl transferase (U/L)	1.023	0.689	0.045	0.628	0.823
**Glucose, serum (mg/dL)**	**4.911**	**2.487**	0.168	2.140	2.742
Iron, refigerated (ug/dL)	1.752	1.443	0.088	1.288	1.559
Lactate dehydrogenase (U/L)	1.050	1.102	0.066	0.973	1.202
Phosphorus (mg/dL)	0.556	0.587	0.040	0.514	0.668
**Total bilirubin (mg/dL)**	**3.366**	**2.555**	0.291	1.841	2.853
Total protein (g/dL)	0.639	0.930	0.064	0.820	1.055
Triglycerides (mg/dL)	0.987	1.285	0.170	1.126	1.867
**Uric acid (mg/dL)**	**2.598**	**2.401**	0.116	2.232	2.675
Sodium (mmol/L)	0.957	0.299	0.034	0.242	0.366
Potassium (mmol/L)	0.654	0.664	0.059	0.559	0.778
**Chloride (mmol/L)**	**2.810**	1.629	0.065	1.460	1.726
Osmolality (mmol/Kg)	0.749	1.165	0.061	1.019	1.263
Globulin (g/dL)	0.501	0.679	0.062	0.529	0.778
Total Cholesterol (mg/dL)	0.457	0.609	0.058	0.488	0.714
Albumin, urine (ug/mL)	1.247	1.459	0.085	1.326	1.680
Creatinine, urine (umol/L)	0.963	0.992	0.108	0.769	1.193
First albumin creatinine ratio (mg/g)	0.723	0.787	0.118	0.636	1.110
**Second albumin (ug/mL)**	0.975	**2.314**	1.184	1.071	5.309
**Second creatinine (mg/dL)**	1.768	**2.056**	0.525	1.459	3.329
**Second albumin creatinine ratio (mg/g)**	1.176	**3.321**	0.825	1.783	5.326
**The volume of urine collection #1**	1.485	**2.082**	0.092	1.948	2.336
**Urine #1 Flow Rate**	**3.262**	**2.921**	0.720	1.743	4.456
Urine osmolality (mOsm/kg)	1.308	1.675	0.179	1.352	2.242
Hepatitis A Antibody	0.031	0.089	0.028	0.046	0.151
Hepatitis B surface antibody	0.034	0.051	0.018	0.023	0.082

Note: Highlighted indicate biomarker selected for univariate logistic regression. Validation on original plus each imputed data set. Random splitting of 60:40 training:validation, λ = 0.001, 50% bagging, 6 maximum number of boosting interactions. Original pseudo-R² = 0.032, imputed data set pseudo-R² ranged from 0.044 to 0.052. Variables selected at this step accounted for more than 50% of the total relative importance: original data was 53.85%; mean of 20 imputation sets was 53.33%.

### STEP 3: Traditional statistical regression results

The training data yielded five biomarkers with significant univariate relationship with depression: hemoglobin (g/dL), Red cell distribution width (%), blood cadmium (nmol/L), cotinine (ng/mL), and total bilirubin (mg/dL) (Table 3). The validation data confirmed the five biomarkers from the training data with the addition of glucose, serum (mg/dL) (Table 3). Since the p-value from the training data was 0.05 for this sixth biomarker it was decided to include this variable in the final model.

**Table 3:** Univariate Logistic Regression statistics.

	TRAINING					VALIDATION				
Biomarker	Odds Ratio	Std. Err.	p-value	CI Low	CI High	Odds Ratio	Std. Err.	p-value	CI Low	CI High
**Hemoglobin (g/dL)**	0.85	0.057	0.042	0.73	0.99	0.85	0.059	0.048	0.72	1.00
Hematocrit (%)	0.95	0.024	0.084	0.90	1.01	0.95	0.025	0.077	0.89	1.01
MCHC (g/dL)	0.85	0.115	0.261	0.63	1.15	0.89	0.131	0.454	0.65	1.23
**Red cell distribution width (%)**	1.20	0.080	0.024	1.03	1.40	1.20	0.079	0.023	1.03	1.40
Platelet count (1000 cells/uL)	1.00	0.002	0.058	1.00	1.01	1.00	0.002	0.083	1.00	1.01
**Blood cadmium (nmol/L)**	1.07	0.018	0.014	1.02	1.11	1.07	0.018	0.012	1.02	1.11
C-reactive protein(mg/dL)	1.18	0.106	0.091	0.97	1.44	1.15	0.098	0.141	0.95	1.38
**Cotinine (ng/mL)**	1.00	0.001	0.011	1.00	1.00	1.00	0.001	0.009	1.00	1.00
Urinary Total NNAL (ng/mL)	1.07	0.092	0.444	0.87	1.32	1.10	0.117	0.390	0.85	1.44
Alkaline phosphotase (U/L)	1.00	0.004	0.197	1.00	1.01	1.00	0.004	0.262	1.00	1.01
Bicarbonate (mmol/L)	0.93	0.045	0.156	0.83	1.03	0.92	0.044	0.133	0.83	1.03
Creatinine (mg/dL)	0.87	0.409	0.770	0.30	2.48	0.87	0.430	0.778	0.28	2.67
**Glucose, serum (mg/dL)**	1.00	0.002	0.050	1.00	1.01	1.01	0.002	0.039	1.00	1.01
**Total bilirubin (mg/dL)**	0.19	0.093	0.009	0.06	0.58	0.24	0.112	0.016	0.08	0.71
Uric acid (mg/dL)	0.97	0.069	0.655	0.83	1.13	0.94	0.062	0.345	0.81	1.08
Chloride (mmol/L)	0.98	0.039	0.700	0.90	1.08	0.98	0.038	0.624	0.90	1.07
Second albumin (ug/mL)	1.00	0.001	0.477	1.00	1.00	1.00	0.001	0.290	1.00	1.00
Second creatinine (mg/dL)	1.00	0.002	0.438	1.00	1.00	1.00	0.001	0.479	1.00	1.00
Second albumin creatinine ratio (mg/g)	1.00	0.000	0.516	1.00	1.00	1.00	0.000	0.315	1.00	1.00
The volume of urine collection #1	1.00	0.001	0.813	1.00	1.00	1.00	0.001	0.651	1.00	1.00
Urine #1 Flow Rate	0.89	0.125	0.438	0.65	1.23	0.86	0.136	0.382	0.60	1.24

Note: Bold Biomarker indicates selection. Multiple imputation logistic regression used taking account of the survey design of NHANES with 15 strata, 31 Primary Sampling Units (PSU).

A multiple logistic regression with these predictors suggested that the effect of hemoglobin was fully mediated by total bilirubin and that cotinine was fully mediated by blood cadmium. Both haemoglobin and cotinine were therefore excluded from the final model, leaving four key biomarkers: red cell distribution width, blood cadmium, serum glucose and total bilirubin.

The direction and significance of the four biomarkers were tested with the combined imputed data sets, allowing for a 50:50 random split between training and validation data. Consistent results were obtained for the multiple logistic regression model in the training and validation data (Table 4).

**Table 4:** Final Four biomarkers from boosted regression.

Biomarker	Training					Validation				
	Odds Ratio	Std. Err.	p-value	95% CI Low	95% CI High	Odds Ratio	Std. Err.	p-value	95% CI Low	95% CI High
Red cell distribution width	1.159	0.079	0.057	0.995	1.350	1.161	0.080	0.063	0.990	1.362
Blood cadmium (nmol/L)	1.060	0.017	0.020	1.015	1.107	1.060	0.017	0.017	1.016	1.106
Glucose, serum (mg/dL)	1.005	0.002	0.066	1.000	1.009	1.005	0.002	0.051	1.000	1.010
Total bilirubin (mg/dL)	0.241	0.112	0.016	0.082	0.703	0.315	0.143	0.034	0.111	0.895
Constant	0.017	0.014	0.001	0.002	0.116	0.012	0.011	0.001	0.002	0.094

Note: Multiple imputation logistic regression using subpopulation based on a random split of approximately 50:50 train:validation (n = 2,590 train: n = 2,637 validation).

The final model controlling for potential confounders and including significant interactions are reported in Table 5.

**Table 5:** Final Multivariate Logistic Regression.

	Odds Ratio	Std. Err.	p-value	95% CI Low	95% CI High
**Biomarkers**					
Red cell distribution width	1.145	0.067	0.037	1.009	1.298
Blood cadmium (nmol/L)	1.024	0.018	0.182	0.987	1.063
Glucose, serum (mg/dL)	1.005	0.002	0.009	1.001	1.008
Total bilirubin (mg/dL)	0.116	0.049	<0.001	0.047	0.284
**Gender**					
Male (Reference)	1.000				
Female	1.610	0.312	0.027	1.064	2.439
**Age group (years)**					
18–34 (Reference)	1.000				
35–44	1.287	0.346	0.364	0.724	2.288
45–54	1.993	0.419	0.005	1.271	3.124
55–64	1.475	0.398	0.171	0.828	2.627
65+	0.660	0.421	0.525	0.169	2.585
**Race**					
Non-Hispanic White (Reference)	1.000				
Mexican Amer/Hispanic	0.409	0.150	0.029	0.186	0.898
Non-Hispanic Black	0.842	0.391	0.718	0.312	2.278
Other	1.552	1.527	0.662	0.189	12.743
**Smoking**					
Never smoked (Reference)	1.000				
Current smoker	0.382	0.162	0.039	0.154	0.946
Former smoker	0.506	0.326	0.308	0.127	2.013
**Food Security**					
Food insecurity (Reference)	1.000				
Full food security	0.492	0.093	0.002	0.328	0.737
**Poverty Income Ratio (PIR)**	0.787	0.058	0.006	0.671	0.923
**BMI Category**					
Normal (Reference)	1.000				
Underweight	2.497	1.630	0.182	0.617	10.101
Overweight	0.864	0.177	0.486	0.557	1.339
Obese	1.007	0.194	0.973	0.666	1.521
**Inactivity**					
Active (Reference)	1.000				
Inactive	1.104	0.135	0.431	0.850	1.433
**Alcohol consumption per month**	1.008	0.014	0.601	0.978	1.038
**Diabetes**					
No (Reference)	1.000				
Yes	0.798	0.210	0.407	0.454	1.403
**Central Nervous System Meds**					
No (Reference)	1.000				
Yes	2.279	0.292	<0.001	1.732	2.999
**Psychotherapeutic Agents**					
No (Reference)	1.000				
Yes	2.912	0.433	<0.001	2.118	4.002
**Interactions:**					
**Age Group by Blood cadmium**					
18–34 (Reference)	1.000				
35–44	0.974	0.024	0.308	0.924	1.027
45–54	0.950	0.019	0.025	0.910	0.993
55–64	0.946	0.029	0.088	0.887	1.009
65+	0.966	0.062	0.606	0.842	1.110
**Diabetes by Blood cadmium**					
No (Reference)	1.000				
Yes	1.115	0.049	0.025	1.016	1.225
**Race by Bilirubin**					
Non-Hispanic White (Reference)	1.000				
Mexican Amer/Hispanic	3.946	1.988	0.016	1.342	11.603
Non-Hispanic Black	1.715	1.333	0.499	0.325	9.050
Other	0.484	0.628	0.585	0.030	7.777
**Smoking by Bilirubin**					
Never smoked (Reference)	1.000				
Current smoker	9.131	4.193	<0.001	3.418	24.398
Former smoker	2.676	2.396	0.290	0.394	18.187
Constant	0.040	0.030	0.001	0.008	0.200

Note: Multiple imputation logistic regression taking account of the complex survey design of NHANES with 15 strata, 31 PSUs. (n = 3,326).

Only Central nervous system medications and / or psychotherapeutic agents and diabetes were included in the final multivariate model. The final validated model, using the original data set of observations including missing values, took account of the complex survey data of NHANES and the test for goodness of fit was not significant (F(9,8) = 2.05, p = 0.163) indicating that the model provided a good fit to the data (76). In addition, the odds ratios and p-values were consistent with the final model in Table 5.

In Table 5, three of the four biomarkers remained significant predictors of depression at the 95% level (p<0.05) after controlling for the potential confounders: red cell distribution width, serum glucose and total bilirubin. Significant interactions were found between bilirubin and the Mexican American / Hispanic group compared to the Non-Hispanic White group, and those who were current smokers compared to those who have never smoked.

Blood cadmium was not found to be significant in the final model (p = 0.180). However, significant interactions (p<0.05) were found between blood cadmium and the 45–54 age group when compared to the younger 18–34 age group, and the diabetic group when compared to the non-diabetic group.

## Discussion

The novel proposed hybrid methodology of fusing data mining techniques using a boosted regression machine learning algorithm with traditional statistical techniques offers an integrated approach to variable selection from a large number of features for large epidemiological studies. The methodology uses a systematic stepped approach that can be applied to large epidemiological population-based studies using complex survey designs.

The methodology ensures that missing data are quantified and addressed using multiple imputation techniques which are appropriate even when the data is not missing at random; however, should multiple imputation not be required, then the methodology is still appropriate using only the original data set. The boosted regression ensures that variable importance is reliably measured taking into account observations that are difficult to predict while at the same time shrinking the effects of these difficult observations in order to avoid over-fitting. This method ensures that multicollinearity does not obscure the influence of important predictors, giving all variables a chance to shine, while taking into account any non-linearity or interaction effects.

An inclusive choice of cut-off values ensures that the regression boosting only reduces the number of predictors from 67 to 21. However, this is enough to remove the multicollinearity problem and to allow a conventional logistic regression analysis that takes account of the complex survey sampling design to be employed in order to further reduce the number of predictors from 21 to 4. Interestingly no biomarker interaction or polynomial terms were found to be significant in the final multivariate logistic regression model making this a particularly simple model which was also found to be robust in relation to important possible confounder variables. Three of the four important biomarkers remained significant to the 95% level despite the inclusion of several confounder covariates and several important biomarker interaction effects with these covariates.

The flexibility of the methodology is exemplified by its ability to take account of any sampling methodology used for an epidemiological study (i.e. simple random samples through to multi-staged complex survey sampling). This was demonstrated using the well-known NHANES data set which used a complex four-staged sampling design encompassing clustered, stratified and weighted data. There are currently no available stepwise or regularized regression procedures which can be applied with complex sample designs making variable selection impossible when there are initially 67 often highly correlated predictors. The boosted regression overcomes this problem by reducing the number of predictors to 21 and ensuring that these predictors are not highly correlated, allowing the complex survey based logistic regression model to be used to manually select the final three predictor variables.

### Validation

There are a number of approaches for assessing models in data mining. This methodology employs the criteria of validity, reliability, parsimony and usefulness to evaluate the effectiveness of the proposed method.

A simulation was initially used to evaluate the effectiveness of the boosted regression and importance percentage metric over a traditional backward stepwise variable selection method to test the impact of a highly correlated predictor in the model such as the case with the biomarker data in this study. A simple random sample of the same size as the original NHANES data was generated (n = 5,227) for six potential predictors. The correlation for two predictors was set to 0.8 to indicate multicollinearity, with the remaining correlations emulated correlations found in the NHANES biomarker data. Two simulations were generated: one with the same betas for all predictors; the other with varying betas. In both simulations the stepwise regression failed to drop one of the two highly correlated predictors, indicating possible estimation instability and structural misspecification [62]. In contrast, the boosted regression downgraded the importance of one of the two highly collinear predictors and produced importance rankings that reflected the beta values used for the other variables. This validates the use of boosted regression for variable selection over stepwise regression.

To further assess the validity of using boosted regression, a lasso regression was used as a comparative machine learning technique for variable selection [63, 64]. This method is similar to boosted regression in terms of its ability to handle a large number of predictors. Using a L1-norm penalty for shrinkage in order to prevent the problem of overfitting, the lasso regression algorithm was employed in the R statistical software using the glmnet package [65]. Consistent with the boosted regression step, the algorithm was performed on the original data and 20 imputed data sets and included cross-validation on 60:40 train:test data. The lasso regression validated the results of the boosted regression analysis but provided a much less parsimonious solution and it was more difficult to combine the variable selection results across the imputed data sets. Results from the lasso analysis contained non-zero coefficients only for the selected variables, making the number of selections a suitable variable importance measure for each variable. Two of the final three biomarkers from the proposed methodology were chosen by the lasso regression in at least 20 of the 21 data sets, and the other was in 16 of the 21 data sets (Table 6). However, for the lasso regression the number of selected variables varied between 18 and 34 for these 21 data sets, with an average of 25 variables selected, making the lasso regression much less parsimonious than the boosted regression method.

**Table 6:** Top 15 biomarkers selected from lasso regression.

Biomarker	Frequency
Blood cadmium (nmol/L)	21
Blood urea nitrogen (mg/dL)	21
**Glucose, serum (mg/dL)**	**21**
Blood lead (ug/dL)	20
Cotinine (ng/mL)	20
**Total bilirubin (mg/dL)**	**20**
Mercury, total (ug/L)	20
Platelet count (1000 cells/uL)	19
Mercury, inorganic (ug/L)	18
Globulin (g/dL)	18
Red blood cell count (million cells/uL)	17
**Red cell distribution width (%)**	**16**
Albumin (g/dL)	16
Phosphorus (mg/dL)	16
Direct HDL-Cholesterol (mg/dL)	15

Note: Bold represents the final 3 biomarkers selected from proposed methodology.

Running a multiple regression directly on the 21 variables selected from the boosted regression on the training and validation data sets, without first using univariate regressions to reduce the number of variables further, produced some unstable results across the training and validation data sets. The odds ratio for Haemoglobin differed drastically between the two models which was consistent with haemoglobin having singularity with haematocrit (r = 0.968) [66]. In addition, predictor significance was inconsistent: total bilirubin was the only significant predictor in the training model and no predictors were significant in the validation model. Finally, the multiple regression models did not highlight mediation effects as significant univariate relationships with the outcome were hidden when all predictors were included in the multivariate models.

Results are considered reliable if the same variables are selected regardless of the data supplied. At step 3 this methodology employs the robust data mining technique of randomly splitting the data into testing and validation data sets at multiple stages of the traditional statistical regression to ensure the variables selected and significance are reliable. In addition, at step 2, validation was performed for each run during the boosted regression by randomly splitting each data set into training and validation sets to make sure that boosting iterations were employed only when improved prediction accuracy was achieved with the validation data set. Usefulness relates to evaluating if the model provides useful information. This hybrid methodology identified three biomarkers associated with depression from an initial set of 67 in the NHANES data set: red cell distribution width, serum glucose, and total bilirubin. The direction of the relationship between depression and the final three biomarkers are broadly concordant with current literature.

Red cell distribution width is a marker of the variability in size of red blood cells or erythrocytes and used for differential diagnosis of anaemia, especially iron deficiency anaemia. This biomarker has been found as a predictor of mortality in the general population [67] and other conditions such as cardiovascular complications [68], Alzheimer’s disease [69] and diabetes [70]. In addition, this biomarker has been investigated as a substitute marker for inflammatory conditions such as inflammatory cancers [71, 72]. Many previous studies have reported the association between inflammatory markers and depression [73, 74], with depression found to be comorbid with both diabetes [25, 75] and cardiovascular diseases [76, 77].

Fasting plasma or serum glucose tests are used to screen for and diagnose diabetes and prediabetes and to monitor for hyperglycaemia or hypoglycaemia. Diabetes is often comorbid with major depression [78, 79], but has a complex bidirectional relationship [75]. An association between depression and glucose utilisation dysfunction has been documented [80].

Bilirubin has been reported to be an antioxidant [81, 82]. Depression has been found to be associated with oxidative stress [83] and mild to moderately elevated serum bilirubin levels are associated with better outcome in diseases involving oxidative stress [82]. The significant interaction found between bilirubin and smoking status is consistent with previous research indicating that smoking is associated with decreased serum bilirubin concentrations [84, 85].

Hemoglobin was found to be fully mediated by total bilirubin. Red blood cells are continuously being broken down, with hemoglobin splitting into globin (protein), iron and heme. The heme initially breaks apart into biliverdin which is reduced to bilirubin. Cotinine was found to be fully mediated by blood cadmium.

### Strengths and Limitations

The strength of this hybrid methodology over other variable selection methods is the potential to adequately handle missing data and complex survey samples using a sound and systematic multi-stepped approach to variable selection. The application of the data mining knowledge discovery process to large epidemiological studies allows researchers to include a large array of data (i.e. variables and observations) to be investigated to generate hypotheses that may have been otherwise overlooked. This is probably true for this NHANES study, particularly in regard to the total bilirubin finding.

The data mining method of splitting data files into training and validation minimises issues of overfitting that is often problematic in traditional statistical techniques with a large number of predictors. The boosted machine learning technique can accommodate different types of variables and has been found to have high predictive accuracy, with shrinkage also used to avoid over-fitting. The iterative learning nature of the algorithm provides researchers with reasonable confidence in the results with its boosted handling of residuals at each iteration. The relative importance measure produced by this technique has been demonstrated to be more effective than the traditional coefficient measures produced by lasso regularized regression and stepwise regression.

A limitation of the methodology is the potential computing power required to perform the machine learning techniques when implementing a small shrinkage parameter. In addition, this study implemented the recommended bagging and number of iterations for the machine learning boosted regression algorithm, but it may be appropriate in the future to run the algorithm on a number of different bagging percentages and number of iterations.

A limitation of this methodology is the complexity of the implementing a multi-stepped variable selection approach compared to a simpler single-stepped variable selection approach. However, unlike simpler single-stepped variable selection procedures such as stepwise regression and regularized regression this multi-stepped method can accommodate missing data using multiple imputation combined with complex survey designs.

The NHANES study used in the example for this hybrid methodology is a large cross-sectional study that contains both missing data and utilises a complex four-stage sampling methodology. The limitations of this type of data restrict the ability to infer the direction of the relationship between the key biomarkers and depression. The self-report instrument of depression, the PHQ-9, may have missed less severe cases of depression [19, 21]. It is also recognised that the imbalance of depressive symptoms in the data set may have resulted in a prediction bias towards the major classes [86].

## Conclusion

An amalgamation of data mining techniques using a machine learning algorithm with traditional statistical techniques provided an effective systematic approach to variable selection in a large epidemiological study, detecting three biomarkers associated with depression for future hypothesis generation: red cell distribution width, serum glucose and total bilirubin. The use of this novel methodology yielded results concordant with previous research, taking account of the missing data and complex survey design of NHANES. This methodology highlights the effectiveness of implementing a hybrid of big data and small sample techniques for variable and potential hypothesis generation.

## Supporting Information

S1 Stata Syntax File Hybrid Methodology Template. (TXT)

## Abbreviations

BMI: Body Mass Index
DIPIT: Data Integration Protocol In Ten-Steps
DSM-IV: Diagnostic and Statistical Manual of Mental Disorder Fourth Edition
FSSM: Food Security Survey Module
MDD: Major Depressive Disorder
MEC: Mobile Examination Center
MART: Multiple Additive Regression Trees
NCHS: National Center for Health Statistics
NHANES: National Health and Nutrition Examination Survey
PHQ-9: Patient Health Questonnaire-9
WHO: World Health Organization (WHO) World Health Organization
